# Ierne, You May Safely Leave the Woman in the Wards

**Published:** 1919-12-06

**Authors:** 


### THE EDITOR'S LETTER-BOX.

[Correspondence on all subjects is invited, but we cannot in any way be responsible for the opinions expressed by our correspondents, who must give their name and address as a guarantee of good faith, but not necessarily for publication. Correspondents are reminded that brevity of style and conciseness of statement greatly facilitate early insertion.]

### "IERNE, YOU MAY SAFELY LEAVE THE WOMAN IN THE WARDS."

Sir,—Long may "Ierne" flourish to encourage or criticise. The "pressing necessities" of the nurse are revealed and public interest is aroused, and is in process of education. "Ierne" grants all that the College has so far achieved, but he want's more, far more, and so do we all.

In looking at the nursing world to-day one thing is clear, to my mind at least: it is that the nurse who has benefited most (from even the little attained) is "the woman in the wards," and entirely because she has behind her the matron and the Hospital Committee.

An evil habit engendered by the war is to label companies of people, either large or small, as good or bad, not to consider them as human entities. "Ierne" appears to recognise matrons as women, they may even have hearts of gold, but Hospital Committees are a race apart—of stone. Surely they are men who have decided to devote their time, experience, and means to the cause of suffering humanity, and they must know that in the hands of the matron and her staff lies much of all they most desire for the welfare of the sick.

In the hospital with which I am connected the matron naturally made it her concern to approach the governors regarding increase of staff, of accommodation, of shorter hours, and better pay, and on the last occasion the College Salaries Report was made the basis of the discussion. I venture to say that this has been the procedure in most hospitals. The advertisements in nursing papers prove that shorter hours and better pay are offered and will improve. Preliminary training-schools and sister-tutors are becoming the rule of the establishment at many hospitals of midwifery, and massage-schools and welfare clinics are valuable additions to the nurses' experience and opportunities.

No, "Ierne," you may safely leave the "woman in the wards," for the time being, to her matron and committee. More pressing problems to solve are the hours and duties of the private nurse, the provision of hotel-hostels if she is to undertake visiting nursing. The pay of the district nurse and midwife and of all who will come under the Health Ministry—provision for these in sickness and distress—the College is at work upon these good objects.

Encourage the nurses to feel that their most reasonable hopes can be fulfilled by combination—united as a strong and effective College of Nursing. Yours, etc.,

14 St. Thomas's Street, S.E. 1, F. A. S.

*December* 3, 1919.

